# Emergency Medicine Cases in Underwater and Hyperbaric Environments: The Use of *in situ* Simulation as a Learning Technique

**DOI:** 10.3389/fphys.2021.666503

**Published:** 2021-05-21

**Authors:** Matteo Paganini, Giulia Mormando, Sandro Savino, Giacomo Garetto, Giulia Tiozzo, Enrico M. Camporesi, Fabrizio Fabris, Gerardo Bosco

**Affiliations:** ^1^Environmental and Respiratory Physiology Laboratory and Master Level II in Diving and Hyperbaric Medicine, Department of Biomedical Sciences, University of Padova, Padua, Italy; ^2^Emergency Medicine, Department of Medicine (DIMED), University of Padova, Padua, Italy; ^3^Department of Medicine (DIMED), University of Padova, Padua, Italy; ^4^ATIP Center for Hyperbaric Medicine, Padua, Italy; ^5^TEAMHealth Research Institute, Tampa General Hospital, Tampa, FL, United States

**Keywords:** diving medicine, hyperbaric medicine, simulation, emergency medicine, SCUBA diving, breath-hold diving, hyperbaric oxygen therapy, environmental medicine

## Abstract

**Introduction:**

Hyperbaric chambers and underwater environments are challenging and at risk of serious accidents. Personnel aiming to assist patients and subjects should be appropriately trained, and several courses have been established all over the world. In healthcare, simulation is an effective learning technique. However, there have been few peer-reviewed articles published in the medical literature describing its use in diving and hyperbaric medicine.

**Methods:**

We implemented the curriculum of the Master’s degree in hyperbaric and diving medicine held at the University of Padova with emergency medicine seminars created by the faculty and validated by external experts. These seminars integrated traditional lectures and eight *in situ* simulation scenarios.

**Results:**

For the hyperbaric medicine seminar, simulations were carried out inside a real hyperbaric chamber at the ATIP Hyperbaric Treatment Centre, only using air and reproducing compression noise without pressurization to avoid damages to the manikins. The four scenarios consisted of hyperoxic seizures, pneumothorax, hypoglycemia, and sudden cardiac arrest. Furthermore, we added a hands-on session to instruct participants to prepare an intubated patient undergoing hyperbaric oxygen treatment with a checklist and simulating the patient transfer inside and outside the hyperbaric chamber. The diving medicine seminar was held at the Y-40 The Deep Joy pool in Montegrotto Terme (Italy), also involving SCUBA/breath-hold diving (BHD) instructors to rescue subjects from the water. These diving medicine scenarios consisted of neurologic syndrome (“taravana/samba”) in BHD, drowning of a breath-hold diver, pulmonary barotrauma in BHD, and decompression illness in a SCUBA diver.

**Conclusion:**

With this experience, we report the integration of simulation in the curriculum of a teaching course in diving and hyperbaric medicine. Future studies should be performed to investigate learning advantages, concept retention, and satisfaction of participants.

## Introduction

Diving and hyperbaric medicine concerns human adaptations and diseases arising in hyperbaric chambers or below the water surface. Several features characterize both settings, and the understanding of physiological responses is fundamental to prevent and treat pathologies developing in such unusual conditions. First, these two environments share an increase of ambient pressure above the atmospheric pressure, causing volume reduction of air-filled cavities and potentially damaging organs such as the lungs and the internal and middle ear (Bosco et al., 2018b). Moreover, the amount of oxygen dissolved into the blood increases while diving and can reach high values when subjects breathe pure oxygen during hyperbaric oxygen therapy (HBOT) (Bosco et al., 2018b). On the other hand, subjects immersed in water can experience critical hypoxia while ascending in breath-hold diving (BHD) (Bosco et al., 2020) or in case of accidents while SCUBA diving. The underwater environment is also notoriously dangerous for the risk of drowning. Several other metabolic and hemodynamic changes make these environments challenging. Therefore, healthcare and technical personnel aiming to assist patients and subjects should be adequately trained, with a strong background in accident prevention and acute response to inherent complications.

In diving and hyperbaric medicine, knowledge is usually delivered through fellowships, master training, or as part of medical residency trainings curriculum. However, diving and hyperbaric medicine is still not an official specialty in most countries, despite recent developments and increasing expertise in the field (Jain, 2017b, pp. 579–582).

Simulation is an effective training modality that has increasingly been adopted by all medicine fields (Issenberg et al., 2005). Boet and colleagues conducted a systematic review investigating the use of simulation in hyperbaric medicine, but, surprisingly, no published material was retrieved, thus remarking the need for research in the field (Boet et al., 2019). Two subsequent papers proposed simulation scenarios focused only on hyperbaric medicine (Sadler et al., 2019; Paetow et al., 2020), but simulation cases in underwater medicine are still lacking. With this work, we describe the introduction of simulation in the curriculum of a Master’s degree course in diving and hyperbaric medicine, providing detailed scenarios along with the available literature to guide actions for the most frequent diseases encountered.

## Methods

The Master’s degree in Hyperbaric and Diving Medicine of the University of Padova is a 12-month-long course, with contents delivered monthly through frontal lectures. In the 2019–2020 edition, we introduced two seminars on emergency medicine cases in diving and hyperbaric medicine, respectively. A list of the most frequent medical emergencies encountered in hyperbaric medicine and water/underwater sports was prepared by four members of the Master’s steering committee (a hyperbaric medicine physician, two emergency medicine physicians, and one anesthesiologist). Eight cases were selected, based on the capability of translating theory into *in situ* simulations: four in the hyperbaric chamber scenario (hyperoxic seizures; pneumothorax; hypoglycemia; sudden cardiac arrest) and four in the water-related environment (ascent “taravana”/syncope in BHD; drowning of a breath-hold diver; lung barotrauma in BHD; decompression illness in a SCUBA diver). Furthermore, a hands-on session was added to the hyperbaric emergency medicine seminar to instruct participants to prepare an intubated patient undergoing HBOT and simulating the transfer inside and outside the chamber.

The scenario logs were prepared by the four experts of the steering committee (specified above) and then reviewed for accuracy by experts in diving and hyperbaric medicine external to the commission, which also provided appropriate final modifications.

### Structure of the Seminars and Materials

On the first day of each seminar, the experts from the course’s steering committee delivered theoretical contents to the students, explaining the peculiar features of fast troubleshooting and early differential diagnosis when facing emergencies in the specific environment. This traditional, 4-h-long lecture was delivered through frontal lectures with a projector’s aid in a classroom. On the second day, two students participated in each scenario, acting as a team, wearing microphones. Before the beginning, the faculty presented the manikin and the equipment to all the students in a unique session. Participants were blind to the cases, proposed randomly among the four prepared for each environment. The scene was broadcasted to a room next to the location via a wireless connection, allowing the trainees not directly involved in the case to hear and watch everything in real time along with physiologic parameters (if players attached a multiparametric monitor to the patient). After each simulated case, a debriefing was conducted by a facilitator from the faculty, involving all the students.

The hyperbaric medicine simulations were carried out at the ATIP Hyperbaric Treatment Centre, Padua (Italy), with the chamber’s doors locked and using air with the compression circuit open to only reproduce compression noise without pressurization, to avoid damages to the manikins. The diving medicine seminar was held at the Y-40 The Deep Joy pool in Montegrotto Terme (Italy), also involving SCUBA/BHD instructors to rescue subjects from the water. The SIMULARTI Simulation Center from the Department of Medicine of the University of Padova (Padova, Italy) provided materials, simulation technicians, and facilitators. A Trauma Hal manikin (model 3040.100, Gaumard Scientific, Miami, FL) or a trained actor (emergency medicine residents from the local residency program) were used for the simulations. Data were recorded by the manikins’ internal sensors using UNI^®^ Unified Simulator Control Software ver 2.41.1.0 (Gaumard Scientific, Miami, FL) but were not collected since the analysis was not among the aims of this work.

### Ethics Statement

The participation in the simulation was voluntary, independent, and without incentives offered. Since no data were collected and there were no risks for the involved subjects, the evaluation was deemed exempt from the local Ethics Committee’s institutional review approval.

## Discussion

It is still difficult to prove that simulation directly improves patients’ outcomes, but a growing body of literature suggests an enhanced acquisition and retention of technical and non-technical skills. Although there are a limited number of studies available on the topic and it is difficult to isolate the effects of simulation, Goldshtein found that the implementation of *in situ* simulation in training programs improves patient morbidity and/or mortality (Goldshtein et al., 2020). Therefore, simulation positively impacts standards of care by improving health professionals’ performance (Harwayne-Gidansky et al., 2020).

Simulation can be used to reproduce various clinical settings, ranging from low-risk, bedside procedures to hazardous and rare clinical events. A procedure performed by a trainee for the first time could be risky in the real environment. The most diffused scenarios include pediatric emergencies, cardiac arrest, or much rarer multi-casualty events in disaster medicine. For these reasons, it would be appropriate to use simulation in diving and hyperbaric medicine.

### The Role of Simulation and Experiential Learning in Diving and Hyperbaric Medicine

Diving and hyperbaric medicine is gaining interest among physicians; however, training is still inconsistent and sometimes inadequate. For example, Jones surveyed 62 physicians of various specialties, finding high percentages of missed indications, including also urgent conditions (Jones, 2017).

Different from traditional teaching methods, simulation can actively involve students, identifying knowledge gaps and potentially prolong concept retention. In this particular case, we used *in situ* simulation to increase the teaching experience’s effectiveness. *In situ* simulation increases individual training and inter-professional collaboration and enhances soft skills such as communication, leadership, and membership. Moreover, *in situ* simulation creates the possibility to simulate in the real environment, providing stressful training in a safe context for high-risk procedures (Martin et al., 2020).

Another stressor highlighted during these simulations was communication. Trainees in the hyperbaric chamber had to communicate outside with the hyperbaric technician using an intercom, with the loud noises of simulated compression/decompression. In the underwater scenario, trainees had to get in touch with the local emergency medical service (EMS) operations center. This critical feature of *in situ* simulation can help trainees, especially in light of recent developments in telemedicine. An advanced telemonitoring system could be tested in these environments and be of help in case of accidents. Patients inside a hyperbaric chamber could also be monitored from the outside, where a senior clinician could help those assisting a critical patient inside, or experts could remotely assist patients diving in austere areas.

*In situ* simulation can also be useful in detecting latent risk threats (Patterson et al., 2013), especially when new protocols, procedures, or devices are introduced in the clinical setting. Such a practice has already been described by Paetow et al. (2020) in clinical hyperbaric medicine settings. Managers can take advantage of *in situ* simulated scenarios for onboarding new staff members, to identify possible problems with new equipment, or to simulate the evacuation of an injured diver by the pre-hospital rescue team from a remote location before a planned diving competition.

The debriefing is the key moment in experiential learning. After each scenario, a facilitator guided all the students through the analysis of actions, errors, decisions, and outcomes to understand different points of view and reach shared solutions to increase the learners’ performance in the future (Sawyer et al., 2016). A debriefing guided by an instructor was preferred over a self-guided one to stimulate self-reflection and maximize learning (Ryoo and Ha, 2015).

In the following sections, each scenario will be discussed, providing a synthesis of recent literature regarding the acute disease’s approach and management. Case logs and descriptions are available as Supplementary Materials.

### Cases in Hyperbaric Medicine

In hyperbaric chambers, ambient pressure is usually increased to 1.5–2.8 atmosphere absolute (ATA) (Leach et al., 1998). During HBOT, subjects inhale 100% oxygen, increasing their blood concentration and producing beneficial effects in tissues. The arterial partial pressure of oxygen (PaO2) can easily reach values above 1300 mmHg in healthy subjects (Clark and Lambertsen, 1971). However, several acute conditions can manifest when these variations exceed physiological adaptation mechanisms.

#### Hyperoxic Seizures

Acute oxygen toxicity in the central nervous system primarily exhibits through generic symptoms such as nausea, vomiting, or sensory nerve alterations, up to generalized tonic–clonic seizure IN its most severe form (Paganini and Camporesi, 2019). The underlying pathophysiology takes into account the increased generation of reactive oxygen species (ROS) and nitric oxide (NO) metabolites, along with metabolic changes, altogether causing membrane damages, regional cerebral blood flow alterations, and neurotransmitter derangements (Jain, 2017a, pp. 84–89). Incidence depends on pressure, concentration of inhaled oxygen, and duration of exposure: the higher the factors, the greater the risk. Current data suggest that this is a rare event during treatments performed below 3 ATA for less than 1 h (Paganini and Camporesi, 2019).

There is no specific guideline on the acute treatment of hyperoxic seizures, but the most reasonable action is the fastest reduction of PaO2 back to physiological values. Therefore, oxygen masks should be removed (when used), and room air should be restored in the pressurized chamber. Then, first aid maneuvers should be enacted to preserve the unconscious patient from falling or striking objects as in generalized seizures, while avoiding the routine use of anticonvulsant drugs. Decompression should be started only after the cessation of seizures to avoid the risk of lung barotrauma. In fact, during convulsions, the glottis is usually closed, and an early or abrupt ascent could lead to gas expansion inside the lungs and potential damages (see next section: *Pneumothorax*). Please see Supplementary Material 1 for the detailed case log.

#### Pneumothorax

At pressure, the lungs are particularly vulnerable due to the fragility of the alveoli. Patients undergoing HBOT should be educated not to hold their breath or close the glottis during decompression since, in that phase, gases in the lungs expand and the amount in excess should escape freely. A spontaneous pneumothorax (PNX) due to air trapping can, however, develop typically during the ascent in predisposed subjects, such as those suffering from acute respiratory distress syndrome (Kot et al., 2008), obstructive pulmonary diseases (e.g., asthma, chronic obstructive pulmonary disease), or asymptomatic carriers of bullae. Overall, the incidence of PNX during HBOT is not clearly established (Cakmak et al., 2015). Symptoms and clinical features of PNX vary based on the severity of the manifestation, ranging from sudden-onset pleuritic chest pain to mild or moderate dyspnea. Signs of small PNX can be subtle, but, when clinically relevant, include diminished breath sounds and subcutaneous emphysema. In the hyperbaric chamber, few instruments can help the diagnosis, and the environmental noise further decreases an already low accuracy of physical examination. Point-of-care, chest ultrasound is a useful tool to diagnose PNX (Soldati et al., 2008) early, but the machine should be approved for the use inside the chamber. If a PNX is suspected, any pressure variation should be halted, the patient should be evaluated, and slow decompression of the chamber should be performed. Once outside, a thorough evaluation at the nearest emergency department (ED) is mandatory after the end of the hyperbaric session for several reasons: (a) patients undergoing HBOT usually have multiple comorbidities, (b) chest pain and dyspnea have a broad differential, and (c) imaging is needed to diagnose the size of PNX and determine further management.

Tension PNX is a life-threatening complication, with an unreliable physical exam (Roberts et al., 2015). Emergent needle chest decompression should be performed without delay in the multiplace chamber if tension PNX is suspected, preferring the fourth or fifth intercostal space anterior to the adults’ midaxillary line to insert an 8-cm over-the-needle large-bore catheter (American College of Surgeons Committee on Trauma, 2018, p. 66). Once outside, further stabilization should be achieved through finger thoracostomy or chest drain insertion performed by an experienced provider, followed by an early transfer to the nearest facility. In the particular case of monoplace chambers, emergent chamber decompression and immediate chest decompression should be performed. Please see Supplementary Material 2 for the detailed case log.

#### Hypoglycemia

Patients suffering from diabetes and complicated by non-healing lower extremity ulcers are among the most frequent populations treated with HBOT. This subset of patients seems to develop hypoglycemia during and after treatments, but the incidence is still unclear. Some studies confirmed the susceptibility of diabetics, especially if insulin-dependent (Ekanayake and Doolette, 2001; Trytko and Bennett, 2003; Al-Waili et al., 2006). The suggested pathophysiology involves increased secretion of insulin and an improved peripheral sensibility to insulin on target organs (Dedov et al., 1987; Wilkinson et al., 2012). On the other hand, Peleg et al. (2013) found no differences in blood sugar levels between diabetics and controls before and after HBOT in a controlled trial. Also, Stevens and colleagues, in a retrospective cohort analysis, found that hypoglycemic events were rare (1.5% on 3136 HBOT sessions), independently associated with Type 1 diabetes, and predicted by a pre-HBOT glucose level of 150 mg/dl (Stevens et al., 2015). Hypoglycemia can manifest with weakness, confusion, and sweating. If suspected, without the need of a stick glucose, plain sugar can be orally administered and the patient carefully evaluated: if improving, the patient can continue the treatment; otherwise, the patient can be decompressed. Please see Supplementary Material 3 for the detailed case log.

#### Cardiac Arrest

Cardiac arrest is a rare event in hyperbaric chambers (Kot, 2014), but a careful evaluation before each session should be carried out by the hyperbaric physician to prevent adverse events, especially for critical patients. Despite the fact that PaO2 levels during HBOT are higher than normal, and tissue survival time is prolonged—the so-called “grace period” (Wright et al., 2016)—basic life support must be started immediately after recognition (Merchant et al., 2020). There is no official recommendation regarding this special environment, but some considerations can be made based on the specific features of multiplace or monoplace chambers (Kot, 2014; Wright et al., 2016).

In the case of multiplace chambers, medical attendants should immediately start CPR while communicating the ongoing situation outside. The provider can consider recruiting other personnel or laypersons to ensure high-quality CPR. Decompression speed should consider the safety of other patients and healthcare personnel inside the chamber. The outer lock can be used to rapidly decompress an arrested patient while ensuring the safety of other people attending the main chamber. An oropharyngeal airway should be placed to avoid air trapping and consequent PNX. On the other hand, in monoplace chambers, the patient should be decompressed as fast as possible to minimize the no-flow time.

Defibrillation can be hazardous inside chambers, and only a few defibrillators have been certified for this purpose. Still, the safest way of delivering shocks seems to be the use of adhesive plates applied to the patient’s chest, connected to an external defibrillator through wires exiting the chamber and activated by a dedicated staff member. To further reduce inflammability, ambient air should be restored inside, a condition easily achieved in multiplace chambers where oxygen is inhaled through masks (Kot, 2014). Please see Supplementary Material 4 for the detailed case log.

#### Intubated Patient Preparation for HBOT

Intubated patients are at increased risk of adverse events both for the severity of their disease and for the high number of medical equipment carried along with them. Particular attention should be paid while transferring a critical patient in a confined space such as hyperbaric chambers, avoiding any tube displacement or circuit/line disconnection. Once inside, the equipment should be accessible and visible at any moment because, during compression, the practitioner will be responsible for the patient’s adaptations. The high complexity of these treatments has been highlighted by several authors (Kot, 2015; Mathieu et al., 2015; Millar, 2015). Future simulation studies could validate a checklist to standardize the transport and medical assistance to intubated patients undergoing HBOT.

### Cases in Underwater Medicine

Changes in environmental pressure and immersion are the particular aspects of underwater physiology. Briefly, the mammal reflex—generated after the face’s immersion—produces bradycardia and blood shift from the peripheral circulation to the thorax, with changes in cardiac pressures. Also, lungs are compressed and PaO2 increases while descending. In SCUBA diving, the stay at depth causes nitrogen retention in tissues, predisposing to bubble formation and decompression illness (DCI) or to depth narcosis (Bosco et al., 2018b). The following cases were selected among the acute diseases related to both BHD and SCUBA diving.

#### Taravana and Ascent Syncope in BHD

In breath-hold divers, PaO_2_ significantly increases due to lung compression and accumulation of O2 in the blood (Bosco et al., 2018a). Conversely, during ascent, the re-expansion of alveoli determines significant hypoxemia (Bosco et al., 2020). The reduced amount of oxygen to the brain can therefore result in neurologic symptoms ranging from focal weaknesses to seizure-like uncontrolled movements (the so-called “taravana” or “samba” phenomenon) or loss of consciousness (also called “shallow water blackout” or “ascent syncope”) (Lindholm and Lundgren, 2009). A breath-hold diver experiencing neurologic symptoms should be placed on high oxygen concentration with a non-rebreather mask and transferred to the nearest ED. When syncope occurs, rescue by a peer and standard basic life support should be enacted to ensure airway patency while transferring the patient on oxygen. Please see Supplementary Material 5 for the detailed case log.

#### Drowning

Drowning is a process resulting in respiratory impairment from submersion or immersion in a fluid, and is a public health issue (van Beeck et al., 2005). Water activities and sports are at particular risk of drowning, especially those performed underwater. In SCUBA diving, drowning occurs mostly after losing consciousness for medical reasons or problems with the air supplies (wrong gas mixtures; equipment failure; entrapment in caves/relicts, and depletion of supplies). Instead, in activities requiring breath-hold, drowning is due to the subjects’ and athletes’ attempt to prolong their time submerged. Drowning is a complication of BHD syncope due to water entering the subject’s unprotected airways while still submerged. Before a breath-hold dive, voluntary hyperventilation has been demonstrated to predispose to ascent syncope—leading to water aspiration—by reducing PaCO_2_. Since hypercarbia has greater influence on the respiratory drive than hypoxemia, voluntary hyperventilation hampers the alarm mechanism giving the subjects a wrong perception of their endurance capability (Lindholm and Lundgren, 2009). Prevention and early recognition are the first steps. The victim should be rescued by trained personnel if the scene if safe. Basic and advanced life support should be performed as early as possible (Szpilman and Morgan, 2020). Please see Supplementary Material 6 for the detailed case log.

#### Lung Barotrauma

Another problem faced during underwater activities is lung volume reduction (“lung squeeze” descending) and re-expansion (ascending) due to the varying environmental pressure. Pulmonary barotrauma usually occurs in SCUBA divers rapidly ascending and holding their breath or when air is trapped inside the lungs (e.g., obstructive pulmonary disease or asthma) (Russi, 1998). In BHD, barotrauma is less frequent since the volume at the end of the dive cannot be larger than it was initially. However, symptoms of barotrauma can range from a simple cough to life-threatening conditions such as pulmonary edema, hemoptysis, PNX, or gas embolism in the worst cases (Ferrigno and Lundgren, 2003). Ferrigno and Lundgren synthesized two possible explanations regarding the pathophysiology of lung damage in breath-hold divers. First, the blood shift experienced underwater causes blood engorgement of pulmonary vessels, thus reducing lung compliance and interfering with re-expansion. Second, compliance could vary across different lung areas, thus predisposing to shear forces development and direct damage or deformation of airways and consequent transient obstruction. Moreover, athletes performing “lung packing” to reach total lung capacity before a dive are particularly predisposed (Ferrigno and Lundgren, 2003). The management of barotrauma in BHD and SCUBA diving should promptly identify and stabilize complications while organizing a timely transfer to the nearest facility. Practitioners should also consider swim-induced pulmonary edema (SIPE) in the differential since this condition has a specific treatment and is potentially fatal (Moon et al., 2016). Please see Supplementary Material 7 for the detailed case log.

#### Decompression Illness

Gases at pressure dissolve in biological liquids and tissues and form bubbles during decompression. Bubbles are composed of inert gases, mostly nitrogen, and can form in tissues or in the bloodstream causing local damage or distal embolization. The manifestations of DCI are broad and non-specific, potentially involving every system. The most frequent presentations are neurologic (e.g., paresthesias/numbness/tingling, focal neurologic deficit), cutaneous (e.g., itching or marbling), or pain in the affected area (Nochetto et al., 2019). Recent activity in the hyperbaric environment (e.g., SCUBA diving, repetitive BHD, HBOT) is fundamental to suspect DCI. The mainstay of treatment is immediate oxygen administration through a non-rebreathing mask and transfer to the nearest hyperbaric facility (Mitchell et al., 2018). Since SCUBA diving is often practiced in remote areas, an urgent transfer by air could be needed. In this case, a pressurized aircraft should be used for long-haul flights, while the maximum recommended fight altitude for helicopters is 300 m (1,000 ft). The standard recommended treatment is the US Navy Treatment Table 6 using oxygen at 2.8 ATA (Naval Sea Systems Command, 2016). Please see Supplementary Material 8 for the detailed case log.

### Limitations

This paper has two main limitations. First, no data regarding perception of the experience or concept retention were collected. Being this experience preliminary and unique, there was no plan to investigate specific indicators. Second, since only a multiplace hyperbaric chamber was available, simulation cases were created for this specific type. Other scenarios should be adapted to monoplace chambers in the future, taking into account the considerations explained in the paper.

## Conclusion

Simulation has gained a fundamental role in healthcare personnel education, proving to have positive effects on training indicators. This work is a primer, proposing eight *in situ* simulation cases in diving and hyperbaric medicine. Further experiences should investigate the effects on training in the near future and propose further scenarios to increase the validity of this teaching technique in the field.

## Data Availability Statement

The original contributions presented in the study are included in the article/Supplementary Material, further inquiries can be directed to the corresponding author/s.

## Author Contributions

MP, GM, SS, FF, and GB contributed to the conception and design of the study. MP, GM, GG, and GB created the cases. EC and FF reviewed the cases for accuracy. MP, GM, SS, GG, GT, and GB organized the sessions. MP wrote the first draft of the manuscript. GM, SS, GT, EC, and GB wrote specific sections of the manuscript. All authors contributed to manuscript revision, read, and approved the submitted version.

## Conflict of Interest

The authors declare that the research was conducted in the absence of any commercial or financial relationships that could be construed as a potential conflict of interest.
