# Supplementary material for: Emergency Medicine Cases in Underwater and Hyperbaric Environments: The Use of in situ Simulation as a Learning Technique
Source: Front Physiol. 2021 May 21;12:666503. doi: 10.3389/fphys.2021.666503 (PMC8176206; doi:10.3389/fphys.2021.666503)
Supplement: Supplementary file 1 [file Data_Sheet_1.PDF]

Scenario Development	
Date of Development:	December 2019 / January 2020
Scenario Developer(s):	Bosco G, Paganini M, Mormando G, Garetto G
Affiliations/Institutions(s):	Department of Biomedical Sciences (DSB) and Department of Medicine, University of Padova (Padova, Italy);<br><br>ATIP Hyperbaric Treatment Center (Padova, Italy)
Contact E-mail:	simulazione.dimed@unipd.it
Last Revision Date:	January 31st, 2020
Revised By:	Fabris F, Camporesi M
Version Number:	1.0

List of abbreviations

ATA: atmospheres absolute

BP: Blood Pressure

CRM: crisis resource management

GCS: Glasgow Coma Scale

HBOT: Hyperbaric Oxygen Therapy

HR: Heart Rate

O2: oxygen

PNX: Pneumothorax

RR: Respiratory Rate

Case Summary 01: A shaky decompression

**Scenario Title:**	**A shaky decompression**
Keywords:	hyperbaric medicine, seizure, hyperoxia, multiplace hyperbaric chamber
Brief Description of Case:	A patient treated in a multiplace hyperbaric chamber for femoral head necrosis has a seizure due to hyperoxia. Learners should stop oxygen and assist the critically ill patient.

**Goals and Objectives**	
Educational Goal:	Management of a complication due to hyperoxia in the hyperbaric chamber.
Medical objectives:	• Recognize condition<br>• Stop 02 flow / restore room air in the chamber<br>• Slow decompression
No CRM objectives	

**Learners, Setting and Personnel**				
Target Learners:	☐ Junior Learners		x Senior Learners	x Staff
	x Physicians	x Nurses	☐ RTS	x Inter-professional

	x Other Learners: Trainees in Diving and Hyperbaric Medicine		
Location:	☐ Sim Lab	x In Situ	☐ Other:
Recommended Number of Facilitators:	Instructors: 2		
	Confederates: 1 hyperbaric technician (outside); 1 patient and 1 facilitator in the chamber		
	Sim Techs: 1		

Initial Patient Information

**Patient Chart**			
Patient Name: Paolo	Age: 65	Gender: M	Weight: 72
No monitor in the multiplace hyperbaric chamber, patient alert, collaborating, not pale, not tachypneic.			
Allergies: none			
Past Medical History: femoral head necrosis	Current Medications: painkillers		

Extra Patient Information

Physical Exam	
Cardio: normal	Neuro: initially asymptomatic; after virtual 30 minutes (reality: 2 minutes), the patient says feeling “like numbness” in the fingers<br><br>Then loss of consciousness and generalized clonic seizure.
Resp: normal	Head & Neck: normal, no trauma
Abdo: normal	MSK/skin: clony
Other: /	

Technical Requirements/Room Vision

Patient
☐ Mannequin *(specify the type and whether infant/child/adult)*
X Standardized Patient
☐ Task Trainer
☐ Hybrid

Special Equipment Required, Required Medications, Moulage
None

Monitors at Case Onset
☐ Patient on a monitor with vitals displayed<br>X Patient not yet on a monitor

Patient Reactions and Exam
• Routinary HBOT session.<br>• Patient has already undergone HBOT, and this is the 4th consecutive session.<br>• Chamber reaches target depth (2.4 ATA), oxygen starts.<br>• Presenting complaint: initially asymptomatic; after virtual 30 minutes (reality: 2 minutes), the patient says feeling like numbness in the fingers.<br>• Then loss of consciousness and generalized clonic seizure.<br>• Trainees suspect hyperoxic seizure, put the patient on the floor, remove oxygen mask, check carotid pulse, and call the technician outside asking to restore ambient air concentrations inside the chamber.<br>• Cessation of seizures after 1 minute; patient still unconscious, breathing heavily<br>• Trainees asks the technician to slowly decompress chamber to surface (to avoid PNX)<br>• While decompressing, the patient progressively regains consciousness (Pain then Verbal)<br>• The patient is carried outside, parameters measured, now Alert

Confederates and Standardized Patients

Confederate and Standardized Patient Roles and Scripts	
Standardized Patient	Initially, the patient is asymptomatic, and while sitting in the hyperbaric chamber, has a seizure.
Confederate	Confederates assist him, and they lay him on the ground and put him in Lateral Safety Position.

Scenario Progression

**Scenario States, Modifiers, and Triggers**				
Patient State/Vitals	Patient Status	Learner Actions, Modifiers & Triggers to Move to Next State		Facilitator Notes
**1. Baseline State**	Initially asymptomatic; after virtual 30 minutes (reality: 2 minutes), the patient says feeling like numbness in the fingers<br><br>Then loss of consciousness and generalized clonic seizure.	<u>Expected Learner Actions:</u><br><br>put the patient on the floor, remove O2 mask, check carotid pulse, call technician outside asking to stop O2 flow and restore ambient air concentrations inside the chamber.	<u>Modifiers and triggers</u><br><br>Stop O2--> seizures stop (2)<br><br>O2 not stopped --> seizures continue (3)	Confederates assist him, and they lay him on the ground and put him in Lateral Safety Position.

**2.**<br><br>FR 18<br><br>GCS: 9 (E2V2M5)	Cessation of seizures after 1 minute; patient still unconscious, breathing heavily.	<u>Expected Learner Actions</u><br><br>Trainees ask the technician to slowly decompress chamber to surface (to avoid PNX) -> end scenario	<u>Modifiers and triggers</u><br><br>Fast decompression --> PNX, stop scenario<br><br>If they don't stop O2 within 2 minutes --> stop scenario.	-
**3.**	Generalized clonic seizure	<u>Expected Learner Actions</u><br><br>put the patient on the floor, remove oxygen mask, check carotid pulse, call technician outside asking to stop oxygen flow and restore ambient air concentrations inside the chamber		

Facilitator Cheat Sheet & Debriefing Tips

- The facilitator asks the team, "How did you feel? What are the emotions you felt?"

- Brief Case Summary

- The facilitator invites the team to produce a "Plus/Delta/Solutions" chart describing: "what went well" (Plus); "what could be improved" (Delta); “what we will do next time” (Solutions).

- To help the team, the facilitator asks questions such as: “What actions or things would you perform again in the same clinical case in reality tomorrow”?

- Address the critical points (e.g., assessing the patient's level of consciousness, decompression when necessary, assessing possible causes of illness, etc.).

- Discuss errors or lack of actions and reflect on the causes to find solutions

- Conclusions on positive things done and answers found to possible errors

References

1. Paganini M, Camporesi E. Side Effects of Hyperbaric Oxygen Therapy. In: Moon RE, ed. Hyperbaric Oxygen Therapy Indications. 14 th ed. North Palm Beach, FL, USA: Best Publishing Company; 2019: 335-339.

2. Jain KK. “Indications, contraindications, and complications of HBO therapy”. In: Jain KK, ed. *Textbook of Hyperbaric Medicine*. 6th ed. Cham: Springer International Publishing AG (2017). p. 89-84.
