# Supplementary material for: Emergency Medicine Cases in Underwater and Hyperbaric Environments: The Use of in situ Simulation as a Learning Technique
Source: Front Physiol. 2021 May 21;12:666503. doi: 10.3389/fphys.2021.666503 (PMC8176206; doi:10.3389/fphys.2021.666503)
Supplement: Supplementary file 2 [file Data_Sheet_2.PDF]

Scenario Development	
Date of Development:	December 2019 / January 2020
Scenario Developer(s):	Bosco G, Paganini M, Mormando G, Garetto G
Affiliations/Institutions(s):	Department of Biomedical Sciences (DSB) and Department of Medicine, University of Padova (Padova, Italy);<br><br>ATIP Hyperbaric Treatment Center (Padova, Italy)
Contact E-mail:	simulazione.dimed@unipd.it
Last Revision Date:	January 31st, 2020
Revised By:	Fabris F, Camporesi M
Version Number:	1.0

List of abbreviations

ATA: atmospheres absolute

BP: Blood Pressure

CRM: crisis resource management

COPD: Chronic Obstructive Pulmonary Disease

GCS: Glasgow Coma Scale

HBOT: Hyperbaric Oxygen Therapy

HR: Heart Rate

O2: oxygen

PNX: Pneumothorax

RR: Respiratory Rate

Case Summary 02: A painful decompression

**Scenario Title:**	**A painful decompression**
Keywords:	Pneumothorax, hyperbaric medicine, multiplace hyperbaric chamber
Brief Description of Case:	A patient treated in a multiplace hyperbaric chamber for difficult healing of osteomyelitis, during decompression, notices sudden sharp right chest pain and dyspnea. Learners should suspect PNX, suspend decompression, and slowly decompress.

**Goals and Objectives**	
Educational Goal:	Management of a complication of decompression in a hyperbaric chamber
Medical Objectives:	Recognize/suspect condition<br>Avoid rapid decompression of the chamber<br>Obtain assistance from outside<br>How to perform emergent PNX needle decompression
No CRM objectives	--

Learners, Setting, and Personnel				
Target Learners:	☐ Junior Learners	X Senior Learners		X Staff
	X Physicians	X Nurses	☐ RTS	X Inter-professional
	X Other Learners: Trainees in Diving and Hyperbaric Medicine			
Location:	☐ Sim Lab	In Situ		☐ Other:
Recommended Number of Facilitators:	Instructors: 2			
	Confederates: 1 hyperbaric technician, 1 patient (mannequin)			
	Sim Techs: 2			

Initial Patient Information

Patient Chart					
Patient Name: Andrea			Age: 71	Gender: M	Weight: 76
No monitor in the multiplace hyperbaric chamber. Patient alert					
Temp: 36 °C	HR: 110 bpm, sinus rhythm	BP: 140/80	RR: 24	O_2Sat: 95%	
Allergies: none					

Past Medical History: active smoker, COPD; treated for difficult healing osteomyelitis to the leg on a previous fracture.	Current Medications: levofloxacin; inhaled b-agonist + ipratropium once daily

Extra Patient Information

**Physical Exam**	
List any pertinent positive and negative findings.	
Cardio: normal	Neuro: normal
Resp: normal, then tachypnea	Head & Neck: normal
Abdo: normal	MSK/skin: normal
Other: /	

Technical Requirements/Room Vision

**Patient**
X Mannequin *(adult)*
☐ Standardized Patient
☐ Task Trainer

☐ Hybrid
**Special Equipment Required, Required Medications, Moulage**
None
**Monitors at Case Onset**
☐ Patient on a monitor with vitals displayed<br>X Patient not yet on a monitor

Scenario Progression

**Scenario States, Modifiers, and Triggers**				
Patient State/Vitals	Patient Status	Learner Actions, Modifiers & Triggers to Move to Next State		Facilitator Notes
**1. Baseline State**<br><br>Rhythm: sinus<br><br>HR: 110<br><br>BP: 140/80<br><br>RR: 24<br><br>O_2SAT: 95%<br><br>T: 36°C<br><br>GCS: 15	A patient in a hyperbaric chamber, during decompression, notices a sudden sharp right chest pain and dyspnea.	<u>Expected Learner Actions</u><br><br>The trainee should suspend decompression.	<u>Modifiers and triggers</u><br><br>Despite these actions, deterioration of PNX: the patient has even more pain and is more tachypneic<br><br>-->progression to 2	-

**2.**Rhythm: sinus<br><br>HR: 120<br><br>BP: 140/80<br><br>RR: 24<br><br>O_2SAT: 95%<br><br>T: 36°C<br><br>GCS: 15	The patient says that the dyspnea is getting worse	Expected Learner Actions<br><br>The trainee should ask for help and ask to put an IV catheter over the needle + disinfectant inside the hyperbaric chamber. Then ask to prepare the emergency trolley and a stretcher ready outside the chamber. Then restart slow decompression, informing the patient.	Modifiers and triggers:<br><br>Despite these actions, patient deteriorates. Level of consciousness decreases to pain (on AVPU scale), the pulse is fast and superficial if checked -> patient is progressing to tension PNX --> go to 3	-
**3.**Rhythm: sinus<br><br>HR: 140<br><br>BP: 80/40<br><br>RR: 30<br><br>O_2SAT: 88%<br><br>T: 36 °C<br><br>GCS: 9	Patient deteriorates. level of consciousness decreases to pain (on AVPU scale), the pulse is fast and superficial if checked.	The trainee should perform needle decompression of tension PNX.	Needle decompression of tension PNX--> resolution of symptoms<br><br>-->progression to 4<br><br>No needle decompression<br><br>-->deterioration of PNX: cardiac arrest → stop scenario if no needle decompression within 1 minute.	
**4.**Rhythm: sinus<br><br>HR: 110<br><br>BP: 140/80<br><br>RR: 24<br><br>O_2SAT: 95%<br><br>T: °C 36<br><br>GCS: 15	Resolution of symptoms, patients become alert again, still feeling pain on the affected site	Expected Learner Actions<br><br>The trainee should ask the technician outside to decompress the hyperbaric chamber slowly.	-<br><br>-	-

Facilitator Cheat Sheet & Debriefing Tips

- The facilitator asks the team, "How did you feel? What are the emotions you felt?"

- Brief Case Summary

- The facilitator invites the team to produce a "Plus/Delta/Solutions" chart describing: "what went well" (Plus); "what could be improved" (Delta); “what we will do next time” (Solutions).

- To help the team, the facilitator asks questions such as: “What actions or things would you perform again in the same clinical case in reality tomorrow”?

- Address the critical points (e.g., assessing the patient's level of consciousness, decompression when necessary, assessing possible causes of illness, etc.).

- Discuss errors or lack of actions and reflect on the causes to find solutions

- Conclusions on positive things done and answers found to possible errors

References

1. Kot J, Michałkiewicz M, Sićko Z. Odma opłucnowa w trakcie leczenia hiperbarycznego [Pneumothorax during hyperbaric oxygenation]. Anestezjol Intens Ter. 2008 Jan-Mar;40(1):35-8.

2. Cakmak T, Battal B, Kara K, Metin S, Demirbas S, Yildiz S, Uzun G. A case of tension pneumothorax during hyperbaric oxygen therapy in an earthquake survivor with crush injury complicated by ARDS (adult respiratory distress syndrome). Undersea Hyperb Med. 2015 Jan-Feb;42(1):9-13.

3. Roberts DJ, Leigh-Smith S, Faris PD, Blackmore C, Ball CG, Robertson HL, Dixon E, James MT, Kirkpatrick AW, Kortbeek JB, Stelfox HT. Clinical Presentation of Patients With Tension Pneumothorax: A Systematic Review. Ann Surg. 2015 Jun;261(6):1068-78. Doi: 10.1097/SLA.0000000000001073.

4. American College of Surgeons. Committee on Trauma. Chapter 4: Thoracic Trauma. In: Advanced trauma life Support® student course manual. ISBN 78-0-9968262-3-5. Tenth Edition. p. 66.
