# Supplementary material for: Emergency Medicine Cases in Underwater and Hyperbaric Environments: The Use of in situ Simulation as a Learning Technique
Source: Front Physiol. 2021 May 21;12:666503. doi: 10.3389/fphys.2021.666503 (PMC8176206; doi:10.3389/fphys.2021.666503)
Supplement: Supplementary file 3 [file Data_Sheet_3.PDF]

Scenario Development	
Date of Development:	December 2019 / January 2020
Scenario Developer(s):	Bosco G, Paganini M, Mormando G, Garetto G
Affiliations/Institutions(s):	Department of Biomedical Sciences (DSB) and Department of Medicine, University of Padova (Padova, Italy);<br><br>ATIP Hyperbaric Treatment Center (Padova, Italy)
Contact E-mail:	simulazione.dimed@unipd.it
Last Revision Date:	January 31st, 2020
Revised By:	Fabris F, Camporesi M
Version Number:	1.0

List of abbreviations

CRM: crisis resource management

GCS: Glasgow Coma Scale

HBOT: Hyperbaric Oxygen Therapy

O2: oxygen

RR: Respiratory Rate

Case Summary 03: A bittersweet decompression

**Scenario Title:**	**A bittersweet decompression**
Keywords:	Hypoglycemia, hyperbaric medicine, multiplace hyperbaric chamber, diabetic foot
Brief Description of Case:	A diabetic patient being treated in a multiplace hyperbaric chamber for foot ulcers experiences hypoglycemia. The learners should recognize symptoms and administer glucose.

**Goals and Objectives**	
Educational Goal:	Management of hypoglycemic crisis
Medical objectives:	Recognize condition<br>Administer sugar by mouth<br>Avoid sudden decompression
No CRM objectives:	

Learners, Setting, and Personnel				
Target Learners:	☐ Junior Learners	x Senior Learners		x Staff
	x Physicians	x Nurses	☐ RTS	x Inter-professional
	x Other Learners: Trainees in Diving and Hyperbaric Medicine			
Location:	☐ Sim Lab	X In Situ		☐ Other:
Recommended Number of Facilitators:	Instructors: 2			
	Confederates: 1 hyperbaric technician, 1 patient			
	Sim Techs: 1			

Initial Patient Information

Patient Chart			
Patient Name: Simona	Age: 73	Gender: F	Weight: 65
Presenting complaint: the patient has a diabetic ulcer			
No monitor in the multiplace hyperbaric chamber, patient alert, collaborating, no pale, no tachypneic.			
Cap glucose: 43 mg/dl	GCS: 15		

Allergies: none	
Past Medical History: Type 2 diabetes complicated by diabetic ulcers.	Current Medications: Insulin, statins, aspirin

Extra Patient Information

**Physical Exam**	
List any pertinent positive and negative findings.	
Cardio: slight tachycardia	Neuro: confused and asthenic patient
Resp: normal	Head & Neck: normal
Abdo: normal	MSK/skin: pale and cold, sweaty skin
Other: diabetic ulcer on the left foot	

Technical Requirements/Room Vision

**Patient**
☐ Mannequin *(specify the type and whether infant/child/adult)*
X Standardized Patient
☐ Task Trainer

☐ Hybrid
**Special Equipment Required, Required Medications, Moulage**
The patient has a diabetic ulcer on the left foot.
**Monitors at Case Onset**
☐ Patient on a monitor with vitals displayed<br>X Patient not yet on a monitor
**Patient Reactions and Exam**
Patient feels confused and very tired

Confederates and Standardized Patients

**Confederate and Standardized Patient Roles and Scripts**	
Standardized patient	A diabetic patient in a hyperbaric chamber has a hypoglycemic crisis. She progressively develops generalized weakness and becomes confused. She says she wants to lie down because she is tired.
Confederate	They help the patient to lay down.

Scenario Progression

Scenario States, Modifiers, and Triggers				
Patient State/Vitals	Patient Status	Learner Actions, Modifiers & Triggers to Move to Next State		Facilitator Notes
**1. Baseline State**<br><br>T: 36°C<br><br>GCS: 15	HBOT started. After virtual 30 minutes (reality: 2 minutes) the patient is progressively confused and profoundly asthenic. She says she wants to lie down because she is tired.	Expected Learner Actions<br><br>The trainees should stop O2 and lay the patient on the ground, start BLS, suspect the cause of the symptoms, and administer glucose by mouth if possible, IV otherwise.	Modifiers and Triggers<br><br>No administration of glucose --> deterioration of neurological status until hypoglycemic coma --> stop scenario<br><br>Administration of glucose --> resolution of symptoms. If the patient gets better, she can continue the hyperbaric therapy.	-

Facilitator Cheat Sheet & Debriefing Tips

- The facilitator asks the team, "How did you feel? What are the emotions you felt?"

- Brief Case Summary

- The facilitator invites the team to produce a "Plus/Delta/Solutions" chart describing: "what went well" (Plus); "what could be improved" (Delta); “what we will do next time” (Solutions).

- To help the team, the facilitator asks questions such as: “What actions or things would you perform again in the same clinical case in reality tomorrow”?

- Address the critical points (e.g., assessing the patient's level of consciousness, decompression when necessary, assessing possible causes of illness, etc.).

- Discuss errors or lack of actions and reflect on the causes to find solutions

- Conclusions on positive things done and answers found to possible errors

References

1. Ekanayake L, Doolette DJ. Effects of hyperbaric oxygen treatment on blood sugar levels and insulin levels in diabetics. SPUMS Journal 2001;31:16-20.5

2. Trytko BE, Bennett MH. Blood sugar changes in diabetic patients undergoing hyperbaric oxygen therapy. SPUMS Journal 2003;33:62-9

3. Al-Waili NS, Butler GJ, Beale J, Abdullah MS, Finkelstein, Merrow M, et al. Influences of hyperbaric oxygen on blood pressure, heart rate and blood glucose levels in patients with diabetes mellitus and hypertension. Arch Med Res. 2006;37:991-7.6

4. Dedov II, Lukich VL, Bol’shakova TD, Gitel EP, Deval AV. Effect of hyperbaric oxygenation on residual insulin secretion in patients with diabetes mellitus type 1. Probl Endocrinol (Mosk). 1987;33(4):10-5.13

5. Wilkinson D, Chapman IM, Heilbronn LK. Hyperbaric oxygen therapy improves peripheral insulin sensitivity in humans. Diabet Med. 2012;29:986-9.

6. Peleg RK, Fishlev G, Bechor Y, Bergan J, Friedman M, Koren S, Tirosh A, Efrati S. Effects of hyperbaric oxygen on blood glucose levels in patients with diabetes mellitus, stroke or traumatic brain injury and healthy volunteers: a prospective, crossover, controlled trial. Diving Hyperb Med. 2013 Dec;43(4):218-21.

7. Stevens SL, Narr AJ, Claus PL, Millman MP, Steinkraus LW, Shields RC, Buchta WG, Haddon R, Wang Z, Murad MH. The incidence of hypoglycemia during HBO2 therapy: A retrospective review. Undersea Hyperb Med. 2015 May-Jun;42(3):191-6.
