# Supplementary material for: Emergency Medicine Cases in Underwater and Hyperbaric Environments: The Use of in situ Simulation as a Learning Technique
Source: Front Physiol. 2021 May 21;12:666503. doi: 10.3389/fphys.2021.666503 (PMC8176206; doi:10.3389/fphys.2021.666503)
Supplement: Supplementary file 4 [file Data_Sheet_4.PDF]

Scenario Development	
Date of Development:	December 2019 / January 2020
Scenario Developer(s):	Bosco G, Paganini M, Mormando G, Garetto G
Affiliations/Institutions(s):	Department of Biomedical Sciences (DSB) and Department of Medicine, University of Padova (Padova, Italy);<br><br>ATIP Hyperbaric Treatment Center (Padova, Italy)
Contact E-mail:	simulazione.dimed@unipd.it
Last Revision Date:	January 31st, 2020
Revised By:	Fabris F, Camporesi M
Version Number:	1.0

List of abbreviations

ACLS: Advanced Cardiovascular Life Support

BLS: Basic Life Support

BP: Blood Pressure

BVM ventilation: Bag-Valve-Mask ventilation

CRM: crisis resource management

CPR: Cardiopulmonary Resuscitation

GCS: Glasgow Coma Scale

HBOT: Hyperbaric Oxygen Therapy

HR: Heart Rate

O2: oxygen

RR: Respiratory Rate

ROSC: Return of Spontaneous Circulation

VF: Ventricular Fibrillation

Case Summary 04: A pounding decompression

**Scenario Title:**	**A pounding decompression**
Keywords:	Cardiac arrest, hyperbaric medicine
Brief Description of Case:	A patient recovering from crush syndrome and mild acute renal failure undergoes another HBOT session in a multiplace hyperbaric chamber. He has been recently transferred from an intensive care unit to a normal ward. The patient suffers a cardiac arrest due to VF in suspected severe hyperkalemia.

**Goals and Objectives**	
Educational Goal:	Management of cardiac arrest in a hyperbaric chamber
Objectives:<br><br>(Medical and CRM)	Recognize condition<br><br>Perform CPR<br><br>Perform decompression<br><br>Obtain assistance from outside the chamber
No CRM objectives:	

Learners, Setting, and Personnel				
Target Learners:	☐ Junior Learners	x Senior Learners		x Staff
	x Physicians	x Nurses	☐ RTS	x Inter-professional
	x Other Learners: Trainees in Diving and Hyperbaric Medicine			
Location:	☐ Sim Lab	x In Situ		☐ Other:
Recommended Number of Facilitators:	Instructors: 2			
	Confederates: 1 hyperbaric technician, one patient (mannequin)			
	Sim Techs: 1			

Initial Patient Information

Patient Chart					
Patient Name: Gianni			Age: 50	Gender: M	Weight: 84
Presenting complaint: Crush syndrome with mild acute renal failure					
Temp: 36 °C	HR: 76 bpm	BP: 120/70	RR: 16	O_2Sat: 100%	FiO_2: AA
Cap glucose: 130 mg/dl			GCS: (E 4 V 5 M 6) 15		

Allergies: none	
Past Medical History: none	Current Medications: hydration, loop diuretics

Extra Patient Information

Physical Exam	
List any pertinent positive and negative findings.	
Cardio: normal	Neuro: normal
Resp: normal	Head & Neck: normal
Abdo: normal	MSK/skin: plaster cast on the left leg
Other: /	

Technical Requirements/Room Vision

**Patient**
X Mannequin *(adult)*
☐ Standardized Patient
☐ Task Trainer
☐ Hybrid
**Special Equipment Required, Required Medications, Moulage**
Wheelchair<br><br>Monitor/defibrillator certified for hyperbaric chamber use.<br><br>Plaster cast on the left leg
**Monitors at Case Onset**
☐ Patient on a monitor with vitals displayed<br><br>X Patient not yet on a monitor
**Patient Reactions and Exam**
While talking with the trainees, the patient asks what to do if he wants to go to the bathroom because "despite drinking a lot, urinary output has been absent more or less for one day."<br><br>Two minutes (virtual 30 minutes) after the beginning of HBOT, the mannequin starts to be confused and then to moan, then unconscious.

Confederates and Standardized Patients

Confederate and Standardized Patient Roles and Scripts	
Confederate	Confirms that the patient doesn’t respond to stimulation and no pulse detected

Scenario Progression

Scenario States, Modifiers, and Triggers				
Patient State/Vitals	Patient Status	Learner Actions, Modifiers & Triggers to Move to Next State		Facilitator Notes
**1. Baseline State**<br><br>Rhythm: sinus<br><br>HR: 76<br><br>BP: 120/70<br><br>RR: 16<br><br>O_2SAT: 100%<br><br>T: 36°C<br><br>GCS: 15	Normal	The trainee inside the chamber talks with the patient, ask for information	None	None

**2.** Rhythm: VF<br><br>BP: ----<br><br>RR: ----<br><br>O_2SAT: ---<br><br>T: 36°C<br><br>GCS 3	Unconscious	Expected Learner Actions<br><br>The learner inside should lay the patient on the ground and recognize cardiac arrest.	Modifiers and Triggers<br><br>- no cardiac arrest recognition → death after 60 seconds (stop scenario)<br><br>- correct cardiac arrest recognition → point 3	
**3.** Rhythm: VF<br><br>BP: ----<br><br>RR: ----<br><br>O_2SAT: ---<br><br>T: 36°C<br><br>GCS 3	Cardiac arrest	Expected Learner Actions<br><br>The learner inside should ask for help outside and start chest compressions. The outside learner should respond and collect information.<br><br>Since the learner inside is alone, he/she asks the outside learner to bring inside expertise and equipment (monitor defibrillator and emergency bag)<br><br>(the confederate inside could suggest this action to the learner outside).	Modifiers and Triggers<br><br>No BLS → death after 60 seconds (stop scenario)<br><br>-BLS → point 4	-
**4.** Rhythm: VF<br><br>BP: ----<br><br>RR: ----<br><br>O_2SAT: ---<br><br>T: 36°C<br><br>GCS 3	Cardiac arrest with VF rhythm	Expected Learner Actions<br><br>The outside learner brings the equipment inside through the outer lock and starts CPR. The learner inside immediately detects VF at the monitor, asks the hyperbaric technician if room air has been restored inside the	Modifiers and Triggers<br><br>- No immediate rhythm recognition or No change of CPR performer → error but continue to the next step<br><br>- ACLS → possible identification of a reversible cause of cardiac arrest and ROSC	

		chamber, and then delivers a shock.<br><br>The learners agree and ask the technician to start decompression while performing CPR:	- Shock delivery without verifying that air room has been restored inside the chamber -> adverse event, fire in the chamber, stop scenario.	
**4.**Rhythm: VF<br><br>BP: ----<br><br>RR: ----<br><br>O_2SAT: ---<br><br>T: 36°C<br><br>GCS 3	Cardiac arrest with VF rhythm	<u>Expected Learner Actions</u><br><br>At the end of decompression, the trainees should bring out the patient and start ACLS protocol with an early check of the rhythm.<br><br>The trainees should verify that emergency medical service has been alerted and discuss potentially reversible causes according to ACLS guidelines	<u>Modifiers and Triggers</u><br><br>- No rhythm check → death and stop scenario<br><br>- ACLS → possible identification of reversible cause of cardiac arrest and ROSC	-
**5.**Rhythm: VF<br><br>BP: ----<br><br>RR: ----<br><br>O_2SAT: ---<br><br>T: 36°C<br><br>GCS 3	Cardiac arrest with VF rhythm	<u>Expected Learner Actions</u><br><br>The learners could check the patient's mock records, where a K+ level of 7 mEq/L is reported that morning or call the ward to obtain clinical information - the confederate will act as the ward's physician telling them that K+ returned 7 mEq/L that morning.	<u>Modifiers and Triggers</u><br><br>• No calcium gluconate administration → refractory VF → asystole → death<br>• Calcium gluconate administration → possible ROSC -> end scenario.	-

Laboratory Results

CBC	Coags
WBC 5,79 10.9/L	INR 1,09
Hgb 134 g/L	aPTT 35 s
Plt 249 10.9/L	
Lytes	
Na 135 mEq/L	
K 7 mEq/L	
Urea 25 mmol/L	
Cr 150 umol/L	

ECGs, X-rays, Ultrasounds, and Pictures

The day before: Color Doppler ultrasound test of lower-limb veins that results negative for deep or peripheral vein thrombosis.

"Subpopliteal, popliteal, and femoral veins normally compressible with preserved flows preserved. No current signs of superficial or deep venous thrombosis in the examined districts."

Facilitator Cheat Sheet & Debriefing Tips

- The facilitator asks the team, "How did you feel? What are the emotions you felt?"

- Brief Case Summary

- The facilitator invites the team to produce a "Plus/Delta/Solutions" chart describing: "what went well" (Plus); "what could be improved" (Delta); "what we will do next time" (Solutions).

- To help the team, the facilitator asks questions such as: "What actions or things would you perform again in the same clinical case in reality tomorrow"?

- Address the critical points (e.g., assessing the patient's level of consciousness, decompression when necessary, assessing possible causes of illness, etc.).

- Discuss errors or lack of actions and reflect on the causes to find solutions

- Conclusions on positive things done and answers found to possible errors

References

1.Merchant RM, Topjian AA, Panchal AR, Cheng A, Aziz K, Berg KM, Lavonas EJ, Magid DJ; Adult Basic and Advanced Life Support, Pediatric Basic and Advanced Life Support, Neonatal Life Support, Resuscitation Education Science, and Systems of Care Writing Groups. Part 1: Executive Summary: 2020 American Heart Association Guidelines for Cardiopulmonary Resuscitation and Emergency Cardiovascular Care. Circulation. 2020 Oct 20;142(16_suppl_2):S337-S357.

2. Kot J. Medical devices and procedures in the hyperbaric chamber. Diving Hyperb Med. 2014 Dec;44(4):223-7.

3. Wright KT, Praske SP, Bhatt NA, Magalhaes RM, Quast TM. Treatment of cardiac arrest in the hyperbaric environment: key steps on the sequence of care--case reports. Undersea Hyperb Med. 2016 Jan-Feb;43(1):71-8.
