# Supplementary material for: Emergency Medicine Cases in Underwater and Hyperbaric Environments: The Use of in situ Simulation as a Learning Technique
Source: Front Physiol. 2021 May 21;12:666503. doi: 10.3389/fphys.2021.666503 (PMC8176206; doi:10.3389/fphys.2021.666503)
Supplement: Supplementary file 5 [file Data_Sheet_5.PDF]

Scenario Development	
Date of Development:	December 2019 / January 2020
Scenario Developer(s):	Bosco G, Paganini M, Mormando G, Garetto G
Affiliations/Institutions(s):	Department of Biomedical Sciences (DSB) and Department of Medicine, University of Padova (Padova, Italy);<br><br>ATIP Hyperbaric Treatment Center (Padova, Italy)
Contact E-mail:	simulazione.dimed@unipd.it
Last Revision Date:	January 31st, 2020
Revised By:	Fabris F, Camporesi M
Version Number:	1.0

List of abbreviations

BP: Blood Pressure

CRM: crisis resource management

DCI: decompression illness

GCS: Glasgow Coma Scale

HBOT: Hyperbaric Oxygen Therapy

HR: Heart Rate

O2: oxygen

RR: Respiratory Rate

EMS operations centre: Emergency Medical Services operations centre

Case Summary 05: Shaky depths

**Scenario Title:**	**Shaky depths**
Keywords:	Seizure, syncope, breath-hold diving
Brief Description of Case:	“Taravana/Samba” in a breath-hold diver

**Goals and Objectives**	
Educational Goal:	Recognition and management of the disease
Medical Objectives:	Recognize condition<br>Administer oxygen<br>Call for help on-site and call EMS Operations Centre.<br>Ask for transfer to the nearest facility with hyperbaric treatment capability.
No CRM objectives	

**Learners, Setting, and Personnel**				
Target Learners:	☐ Junior Learners		Χ Senior Learners	x Staff
	x Physicians	x Nurses	☐ RTS	Χ Inter-professional

	x Other Learners: Trainees in Diving and Hyperbaric Medicine		
Location:	☐ Sim Lab	x In Situ	☐ Other:
Recommended Number of Facilitators:	Instructors: 2		
	Confederates: 1 divemaster, one breath-hold diver, one bystander		
	Sim Techs: 1		

Initial Patient Information

Patient Chart			
Patient Name: Gianluca	Age: 27	Gender: M	Weight: 68
Presenting complaint: neurologic syndrome after re-emersion from a breath-hold dive			
GCS: (E3 V4 M6) 13			
Allergies: not known			
Past Medical History: no diseases	Current Medications: none		

Extra Patient Information

Physical Exam	
Cardio: normal	Neuro: confused but follow simple commands, open eyes when called, right leg shaking
Resp: normal	Head & Neck: normal
Abdo: normal	MSK/skin: normal
Other: /	

Technical Requirements/Room Vision

Patient
☐ Mannequin *(specify the type and whether infant/child/adult)*
X Standardized Patient
☐ Task Trainer
☐ Hybrid
**Special Equipment Required, Required Medications, Moulage**
Mobile phone<br>Oxygen cylinder and non-rebreathing mask

Swimsuit
**Monitors at Case Onset**
☐ Patient on a monitor with vitals displayed<br>X Patient not yet on a monitor
**Patient Reactions and Exam**
Right leg shaky, then stops but numbness and motor deficit.

Confederates and Standardized Patients

**Confederate and Standardized Patient Roles and Scripts**	
Divemaster	Supportive - He rescues the diver from water, asks to bring O2 and calls EMS

Scenario Progression

Scenario States, Modifiers, and Triggers				
Patient State/Vitals	Patient Status	Learner Actions, Modifiers & Triggers to Move to Next State		Facilitator Notes
**1. Baseline State**<br><br>GCS: 13	Rescued by the divemaster. Confused but follow simple commands, open eyes when called, right leg shaking	<u>Expected Learners Actions</u><br><br>• Ask what happened to the divemaster<br>• Ask the patient's symptoms and past medical history.<br>• Suspect "taravana" syndrome and ask for oxygen cylinder + non-rebreathing mask	<u>Modifiers and Triggers</u><br><br>Ask for oxygen → continue to point 2<br><br>Not oxygen → stop scenario after 3 minutes.	-
**2.**	Less confused with oxygen, stop shaking, says feeling numbness and motor deficit in the right leg.	<u>Expected Learners Actions</u><br><br>• Confirm taravana with the possible complication of DCI<br>• A phone call to EMS operations center<br>• Ask for a transfer to an ED with HBOT capability.	<u>Modifiers and Triggers</u><br><br>End scenario	-

Facilitator Cheat Sheet & Debriefing Tips

- The facilitator asks the team, "How did you feel? What are the emotions you felt?"

- Brief Case Summary

- The facilitator invites the team to produce a "Plus/Delta/Solutions" chart describing: "what went well" (Plus); "what could be improved" (Delta); “what we will do next time” (Solutions).

- To help the team, the facilitator asks questions such as: “What actions or things would you perform again in the same clinical case in reality tomorrow”?

- Address the critical points (e.g., assessing the patient's level of consciousness, decompression when necessary, assessing possible causes of illness, etc.).

- Discuss errors or lack of actions and reflect on the causes to find solutions

- Conclusions on positive things done and answers found to possible errors

References

1. Bosco G, Rizzato A, Martani L, Schiavo S, Talamonti E, Garetto G, Paganini M, Camporesi EM, Moon RE. Arterial Blood Gas Analysis in Breath-Hold Divers at Depth. Front Physiol. 2018 Nov 5;9:1558. doi: 10.3389/fphys.2018.01558.

2. Lindholm P, Lundgren CE. The physiology and pathophysiology of human breath-hold diving. J Appl Physiol (1985). 2009 Jan;106(1):284-92. Doi: 10.1152/japplphysiol.90991.2008. Epub 2008 Oct 30. PMID: 18974367.
