# Supplementary material for: Emergency Medicine Cases in Underwater and Hyperbaric Environments: The Use of in situ Simulation as a Learning Technique
Source: Front Physiol. 2021 May 21;12:666503. doi: 10.3389/fphys.2021.666503 (PMC8176206; doi:10.3389/fphys.2021.666503)
Supplement: Supplementary file 6 [file Data_Sheet_6.PDF]

Scenario Development	
Date of Development:	December 2019 / January 2020
Scenario Developer(s):	Bosco G, Paganini M, Mormando G, Garetto G
Affiliations/Institutions(s):	Department of Biomedical Sciences (DSB) and Department of Medicine, University of Padova (Padova, Italy);<br><br>ATIP Hyperbaric Treatment Center (Padova, Italy)
Contact E-mail:	simulazione.dimed@unipd.it
Last Revision Date:	January 31st, 2020
Revised By:	Fabris F, Camporesi M
Version Number:	1.0

List of abbreviations

ACLS: Advanced Cardiovascular Life Support

AED: Automated External Defibrillator

BLS: Basic Life Support

BP: Blood Pressure

BVM ventilation: Bag-Valve-Mask ventilation

CPR: Cardiopulmonary Resuscitation

CRM: Crisis Resource Management

EMS operations centre: Emergency Medical Services operations centre

GCS: Glasgow Coma Scale

HR: Heart Rate

IO access: Intraosseous access

IV: intravenous access

O2: oxygen

ROSC: Return of Spontaneous Circulation

RR: Respiratory Rate

VF: Ventricular Fibrillation

Case Summary 06: Floating in the thermal pool

**Scenario Title:**	**Floating in the thermal pool**
Keywords:	Drowning; cardiac arrest; breath-hold diving;
Brief Description of Case:	A breath-hold diver is training for an international competition. He is found unconscious underwater after ascent syncope.

**Goals and Objectives**	
Educational Goal:	Recognition and management of the disease
Medical Objectives:	Recognize condition<br>Perform CPR<br>Follow ACLS guidelines
No CRM Objectives	

Learners, Setting and Personnel				
Target Learners:	☐ Junior Learners		x Senior Learners	☐ Staff
	x Physicians	x Nurses	☐ RTS	x Inter-professional
	x Other Learners: Trainees in Diving and Hyperbaric Medicine			
Location:	☐ Sim Lab		x In Situ	☐ Other:
Recommended Number of Facilitators:	Instructors: 2			
	Confederates: 1 divemaster, 1 patient			
	Sim Techs: 1			

Initial Patient Information

Patient Chart					
Patient Name: Marco			Age: 27	Gender: M	Weight: 75
Presenting complaint: cardiac arrest after drowning					
Temp: 33	HR: --	BP: --	RR: --	O_2Sat: --	FiO_2: --
Allergies: Not known					

Past Medical History: unknown	Current Medications: unknown

Extra Patient Information

**Physical Exam**	
List any pertinent positive and negative findings.	
Cardio: no pulse	Neuro: unresponsive
Resp: no breaths	Head & Neck: --
Abdo: ---	MSK/skin: pale, wet
Other: /	

Technical Requirements/Room Vision

**Patient**
X Mannequin *(adult)*
☐ Standardized Patient
☐ Task Trainer

☐ Hybrid
**Special Equipment Required, Required Medications, Moulage**
EMS clothes<br>AED<br>Monitor defibrillator<br>Emergency bag<br>Suction apparatus<br>Adrenaline, Amiodarone, Normal Saline<br>Swimsuit
**Monitors at Case Onset**
☐ Patient on a monitor with vitals displayed<br>☓ Patient not yet on a monitor
**Patient Reactions and Exam**
The skin is wet.

Confederates and Standardized Patients

**Confederate and Standardized Patient Roles and Scripts**	
Divemaster	He is agitated, anxious while performing CPR. AED is on-site, but it's still not placed. He rescued the patient immediately after he lost consciousness underwater and immediately started BLS. The divemaster says that "he was training for an international breath-hold diving competition to be held in Croatia

	the next month. Today he was training on buccal pumping and lung packing. I know he was pushing himself to the limit…!”

Scenario Progression

Scenario States, Modifiers, and Triggers				
Patient State/Vitals	Patient Status	Learner Actions, Modifiers & Triggers to Move to Next State		Facilitator Notes
**1. Baseline State**<br><br>Rhythm: PEA<br><br>HR: 30<br><br>BP: --<br><br>RR: --<br><br>O_2SAT: % --<br><br>T: 33°C<br><br>GCS: 3	*Cardiac arrest*	Expected Learner Actions<br><br>Check pulse and ventilation; confirm cardiac arrest.<br><br>One trainee changes CPR performer, the other checks rhythm -> they recognize PEA -> no shock indicated -> restart CPR	Modifiers and Triggers<br><br>During CPR, if they do not confirm the cardiac arrest or do not check rhythm or do not change CPR performer: error but next step<br><br>All ok: next step<br><br>No CPR → stop scenario, death	The divemaster already provided Ventilations after the rescue from water.
**2.**<br><br>Rhythm: PEA<br><br>HR: 30<br><br>BP: --<br><br>RR: --<br><br>O_2SAT: % --<br><br>T: 33°C<br><br>GCS: 3		Expected Learner Actions<br><br>CPR 30:2 (BVM with 100% O2) Peripheral venous access or IO access obtained. Adrenaline 1 mg administration	Modifiers and Triggers<br><br>If more than 3 peripheral venous access attempts failed without going to IO, or BVM ventilation not provided, → stop the scenario<br><br>Correct -> next step	

**3.**<br><br>Rhythm: PEA<br><br>HR: 30<br><br>BP: --<br><br>RR: --<br><br>O_2SAT: % --<br><br>T: 33°C<br><br>GCS: 3		<u>Expected Learner Actions</u><br><br>Check rhythm/pulse after 2 minutes -> PEA -> CPR continued<br><br>Provide adequate BVM ventilation, ongoing 30:2 CPR<br><br>Stabilize airway: intubation or supraglottic airway device	<u>Modifiers and Triggers</u><br><br>If they don't provide the right sequence --> error but next step	
**4.**<br><br>Rhythm: FV<br><br>HR: --<br><br>BP: --<br><br>RR: --<br><br>O_2SAT: % --<br><br>T: 33°C<br><br>GCS: 3		<u>Expected Learner Actions</u><br><br>Adequate ventilation through airway established<br><br>Check rhythm after 2 minutes: VF -> shock delivered -> restart CPR	<u>Modifiers and Triggers</u><br><br>- check pulse after shock delivery: error but next step	
**5.**<br><br>Rhythm: sinus rhythm<br><br>HR: 80<br><br>BP: 70/40<br><br>RR: 18<br><br>O_2SAT: 100%<br><br>T: 33°C		<u>Expected Learner Actions</u><br><br>Ongoing CPR<br><br>Check rhythm and pulse after 2 minutes: ROSC - pulse present, organized rhythm present.<br><br>Post ROSC care	End scenario	

GCS: 3		Contact EMS Operations center, ask for transfer.		

Facilitator Cheat Sheet & Debriefing Tips

- The facilitator asks the team, "How did you feel? What are the emotions you felt?"

- Brief Case Summary

- The facilitator invites the team to produce a "Plus/Delta/Solutions" chart describing: "what went well" (Plus); "what could be improved" (Delta); “what we will do next time” (Solutions).

- To help the team, the facilitator asks questions such as: “What actions or things would you perform again in the same clinical case in reality tomorrow”?

- Address the critical points (e.g., assessing the patient's level of consciousness, decompression when necessary, assessing possible causes of illness, etc.).

- Discuss errors or lack of actions and reflect on the causes to find solutions

- Conclusions on positive things done and answers found to possible errors

References

1.Lindholm P, Lundgren CE. The physiology and pathophysiology of human breath-hold diving. J Appl Physiol (1985). 2009 Jan;106(1):284-92. Doi: 10.1152/japplphysiol.90991.2008. Epub 2008 Oct 30. PMID: 18974367.

2. Beeck EF van, Branche CM, Szpilman D, Modell JH, Bierens JJLM. A new definition of drowning: towards documentation and prevention of a global public health problem. Bull World Health Organ 2005;83(11):853–856.

3. Szpilman D, Morgan P. Management for the Drowning Patient. Chest. 2020 Oct 14:S0012-3692(20)34896-0. doi: 10.1016/j.chest.2020.10.007.
