# Supplementary material for: Emergency Medicine Cases in Underwater and Hyperbaric Environments: The Use of in situ Simulation as a Learning Technique
Source: Front Physiol. 2021 May 21;12:666503. doi: 10.3389/fphys.2021.666503 (PMC8176206; doi:10.3389/fphys.2021.666503)
Supplement: Supplementary file 7 [file Data_Sheet_7.PDF]

Scenario Development	
Date of Development:	December 2019 / January 2020
Scenario Developer(s):	Bosco G, Paganini M, Mormando G, Garetto G
Affiliations/Institutions(s):	Department of Biomedical Sciences (DSB) and Department of Medicine, University of Padova (Padova, Italy);<br><br>ATIP Hyperbaric Treatment Center (Padova, Italy)
Contact E-mail:	simulazione.dimed@unipd.it
Last Revision Date:	January 31st, 2020
Revised By:	Fabris F, Camporesi M
Version Number:	1.0

List of abbreviations

BHD: Breath-Hold Divers

BP: Blood Pressure

CRM: Crisis Resource Management

ED: Emergency Department

EMS operations centre: Emergency Medical Services operations centre

GCS: Glasgow Coma Scale

HR: Heart Rate

O2: oxygen

RR: Respiratory Rate

Case Summary 07: A breathtaking dive

**Scenario Title:**	**A breathtaking dive**
Keywords:	Pulmonary edema; hemoptysis; lung barotrauma; breath-hold diving; diving medicine; SCUBA diving;
Brief Description of Case:	A patient ascending after a breath-hold dive is intensely coughing at the poolside, and then has hemoptysis. The learners suspect lung barotrauma.

**Goals and Objectives**	
Educational Goal:	Recognition and management of the disease
Objectives:<br>(Medical and CRM)	Recognize<br>Provide oxygen<br>Transfer the patient to the ED
EPAs Assessed:	

**Learners, Setting and Personnel**				
Target Learners:	☐ Junior Learners		x Senior Learners	☐ Staff
	x Physicians	x Nurses	☐ RTS	x Inter-professional

	x Other Learners: Trainees in Diving and Hyperbaric Medicine		
Location:	☐ Sim Lab	x In Situ	☐ Other:
Recommended Number of Facilitators:	Instructors: 2		
	Confederates: 1 divemaster		
	Sim Techs: 1		

Initial Patient Information

Patient Chart			
Patient Name: Silvio	Age: 32	Gender: M	Weight: 70
Presenting complaint: intense coughing, GCS: 15 (E 4 V 5 M 6)			
Allergies: None			
Past Medical History: had nose bleeding after a breath-hold dive a few years ago.	Current Medications: None		

Extra Patient Information

Physical Exam	
Cardio: normal	Neuro: normal
Resp: bilateral cracklings, especially at apexes	Head & Neck: normal
Abdo: normal	MSK/skin: normal
Other: normal	

Technical Requirements/Room Vision

Patient
X Mannequin *(adult)*
☐ Standardized Patient
☐ Task Trainer
☐ Hybrid
**Special Equipment Required, Required Medications, Moulage**
Oxygen Cylinder + non-rebreathing mask<br>Present mock blood near the mouth and on a tissue in the mannequin's hand.

Monitors at Case Onset
☐ Patient on a monitor with vitals displayed<br><br>X Patient not yet on a monitor
**Patient Reactions and Exam**
The patient was learning a new BHD technique: "A friend told me about lung packing, so today I tried to inflate my lungs as he explained, using buccal pumping. I don't understand what's happening now." His words are frequently interrupted by cough.<br><br>*When asked to inspire deeply, the cough worsens for a brief amount of time.*<br><br>He feels better on oxygen

Confederates and Standardized Patients

Confederate and Standardized Patient Roles and Scripts	
Dive Master	*Supportive - provide oxygen if not suggested by the learners, provides a saturimeter he has in his locker.*

Scenario Progression

Scenario States, Modifiers, and Triggers				
Patient State/Vitals	Patient Status	Learner Actions, Modifiers & Triggers to Move to Next State		Facilitator Notes
**1. Baseline State**<br><br>Rhythm: --<br><br>HR: --<br><br>BP: 130/80<br><br>RR: 25<br><br>O_2SAT: 91 %<br><br>T: --°C<br><br>GCS: 15	*Alert, tachypneic*	Expected Learner Actions<br><br>The trainees ask for information, ask for the first rescue kit and oxygen<br><br>Saturimeter is brought by the divemaster<br><br>Provide oxygen through a non-rebreathing mask	Modifiers and Triggers<br><br>No oxygen -> stop scenario after 3 minutes	-
**2.**<br><br>BP: 130/80<br><br>RR 20<br><br>Sat 95%		Expected Learner Actions<br><br>Saturation increases, symptoms improve.<br>Blood cuff and stethoscope are provided.<br>BP is normal, lung exam abnormal.<br>Call EMS and ask for a transfer to the ED	Modifiers and Triggers	-

Facilitator Cheat Sheet & Debriefing Tips

- The facilitator asks the team, "How did you feel? What are the emotions you felt?"

- Brief Case Summary

- The facilitator invites the team to produce a "Plus/Delta/Solutions" chart describing: "what went well" (Plus); "what could be improved" (Delta); “what we will do next time” (Solutions).

- To help the team, the facilitator asks questions such as: “What actions or things would you perform again in the same clinical case in reality tomorrow”?

- Address the critical points (e.g., assessing the patient's level of consciousness, decompression when necessary, assessing possible causes of illness, etc.).

- Discuss errors or lack of actions and reflect on the causes to find solutions

- Conclusions on positive things done and answers found to possible errors

References

1.Russi EW. Diving and the risk of barotrauma. Thorax. 1998 Aug;53 Suppl 2(Suppl 2):S20-4.

2. Ferrigno M, Lundgren CE. Breath-hold Diving. In: Brubakk AO, Neuman TS, eds. Bennett and Elliott’s Physiology and Medicine of Diving. 5th ed. Edinburgh: Saunders; 2003: 153-180.

3. Moon RE, Martina SD, Peacher DF, Potter JF, Wester TE, Cherry AD, Natoli MJ, Otteni CE, Kernagis DN, White WD, Freiberger JJ. Swimming-Induced Pulmonary Edema: Pathophysiology and Risk Reduction With Sildenafil. Circulation. 2016 Mar 8;133(10):988-96.
