# Supplementary material for: Emergency Medicine Cases in Underwater and Hyperbaric Environments: The Use of in situ Simulation as a Learning Technique
Source: Front Physiol. 2021 May 21;12:666503. doi: 10.3389/fphys.2021.666503 (PMC8176206; doi:10.3389/fphys.2021.666503)
Supplement: Supplementary file 8 [file Data_Sheet_8.PDF]

Scenario Development	
Date of Development:	December 2019 / January 2020
Scenario Developer(s):	Bosco G, Paganini M, Mormando G, Garetto G
Affiliations/Institutions(s):	Department of Biomedical Sciences (DSB) and Department of Medicine, University of Padova (Padova, Italy);<br><br>ATIP Hyperbaric Treatment Center (Padova, Italy)
Contact E-mail:	simulazione.dimed@unipd.it
Last Revision Date:	January 31st, 2020
Revised By:	Fabris F, Camporesi M
Version Number:	1.0

List of abbreviations

CRM: Crisis Resource Management

DCI: Decompression Illness

ED: Emergency Department

EMS: Emergency Medical Services

GCS: Glasgow Coma Scale

HBOT: Hyperbaric Oxygen Therapy

O2: oxygen

RR: Respiratory Rate

Case Summary 08: decompression illness

Scenario Title:	A thousand blue bubbles
Keywords:	Decompression illness; decompression sickness; SCUBA diving; hyperbaric medicine; diving medicine;
Brief Description of Case:	A SCUBA diver does not follow decompression targets during re-emersion. While collecting his stuff, the diver starts feeling his left leg strange. A suspect diagnosis of decompression illness is made, and the patient transferred to the nearest hyperbaric facility.

Goals and Objectives	
Educational Goal:	Recognition and management of the disease
Medical Objectives:	Recognize DCI<br>Provide oxygen<br>Ask for a transfer to a hyperbaric chamber
No CRM objectives	

<table>
<tr><th colspan="5">Learners, Setting, and Personnel</th></tr>
<tr><td rowspan="3">Target Learners:</td><td colspan="2">☐ Junior Learners</td><td>x Senior Learners</td><td>☐ Staff</td></tr>
<tr><td>☓ Physicians</td><td>x Nurses</td><td>☐ RTS</td><td>☓ Inter-professional</td></tr>
<tr><td colspan="4">x Other Learners: Trainees in Diving and Hyperbaric Medicine</td></tr>
<tr><td>Location:</td><td colspan="2">☐ Sim Lab</td><td>x In Situ</td><td>☐ Other:</td></tr>
<tr><td rowspan="3">Recommended Number of Facilitators:</td><td colspan="4">Instructors: 2</td></tr>
<tr><td colspan="4">Confederates: 1 divemaster, 1 patient</td></tr>
<tr><td colspan="4">Sim Techs: 1</td></tr>
</table>

Initial Patient Information

<table>
<tr><th colspan="4">Patient Chart</th></tr>
<tr><td>Patient Name: Marco</td><td>Age: 65</td><td>Gender: M</td><td>Weight: 75</td></tr>
<tr><td colspan="4">Presenting complaint: weakness and paresthesia in the left leg</td></tr>
<tr><td colspan="4">Allergies: None</td></tr>
</table>

Past Medical History: Hypertension	Current Medications: angiotensin receptor blocker, antiaggregant

Extra Patient Information

**Physical Exam**	
Cardio: normal	Neuro: left leg: sensation reduced on the feet, limb weakness (not able to rise against the examiner's strength)
Resp: normal	Head & Neck: normal
Abdo: normal	MSK/skin: normal
Other: /	

Technical Requirements/Room Vision

**Patient**
☐ Mannequin *(specify the type and whether infant/child/adult)*
X Standardized Patient
☐ Task Trainer
☐ Hybrid

Special Equipment Required, Required Medications, Moulage
Oxygen cylinder + non-rebreathing mask<br><br>SCUBA suit and mask on the subject, SCUBA equipment on site.
**Monitors at Case Onset**
☐ Patient on a monitor with vitals displayed<br><br>X Patient not yet on a monitor
**Patient Reactions and Exam**
Stable, he knows what's happening – having studied it during the diving training. Asks for information regarding the transfer and where and expresses concerns for his family.

Confederates and Standardized Patients

Confederate and Standardized Patient Roles and Scripts	
Standardized Patient	The patient says, "I progressively started feeling tingling on my left foot, then like numb, and now I can't walk properly. My left leg is weaker than normal..."
Dive Master	Describes situation and O2 availability on site

Scenario Progression

<table>
<tr><th colspan="5">Scenario States, Modifiers, and Triggers</th></tr>
<tr><td>Patient State/Vitals</td><td>Patient Status</td><td colspan="2">Learner Actions, Modifiers & Triggers to Move to Next State</td><td>Facilitator Notes</td></tr>
<tr><td>1. Baseline State

RR: 16

GCS: 15</td><td>Alert, simulating numbness and weakness in the left leg</td><td><u>Expected Learner Actions</u>

Trainees ask for information, symptoms.
Suspect DCI
Ask for and administer O2</td><td><u>Modifiers and Triggers</u>

If they don't administer O2, → stop the scenario

If they administer O2 → continue to step 2</td><td>-</td></tr>
<tr><td>2.</td><td>Alert, simulating numbness and weakness in the left leg</td><td><u>Expected Learner Actions</u>

Call EMS operations center

Ask to transfer the patient to the nearest hyperbaric chamber for HBOT.</td><td><u>Modifiers and Triggers</u>

Stop scenario</td><td>-</td></tr>
</table>

Facilitator Cheat Sheet & Debriefing Tips

- The facilitator asks the team, "How did you feel? What are the emotions you felt?"

- Brief Case Summary

- The facilitator invites the team to produce a "Plus/Delta/Solutions" chart describing: "what went well" (Plus); "what could be improved" (Delta); “what we will do next time” (Solutions).

- To help the team, the facilitator asks questions such as: “What actions or things would you perform again in the same clinical case in reality tomorrow”?

- Address the critical points (e.g., assessing the patient's level of consciousness, decompression when necessary, assessing possible causes of illness, etc.).

- Discuss errors or lack of actions and reflect on the causes to find solutions

- Conclusions on positive things done and answers found to possible errors

References

1. Nochetto M, Nord D, Saraiva C, et al. Diving Injuries. In: Denoble PJ, ed. DAN Annual Diving Report 2019 Edition: A report on 2017 diving fatalities, injuries, and incidents. Durham (NC): Divers Alert Network; 2019. Section 3. Available from: https://www.ncbi.nlm.nih.gov/books/NBK562524/

2. Mitchell SJ, Bennett MH, Bryson P, Butler FK, Doolittle DJ, Holm JR, Kot J, Lafère P. Consensus guideline: Pre-hospital management of decompression illness: expert review of key principles and controversies. Undersea Hyperb Med. 2018 May-Jun;45(3):273-286.

3. Naval Sea Systems Command. US Navy Diving Manual. Revision 7. Washington, DC: US Government Printing Office; December 1, 2016. Accessed: February 4 th , 2021. Available at: https://www.navsea.navy.mil/Portals/103/Documents/SUPSALV/Diving/US%20DIVING%20MANUAL_REV7.pdf?ver=2017-01-11-102354-393
